# Extraction and Analysis of Gigantol from *Dendrobium officinale* with Response Surface Methodology

**DOI:** 10.3390/molecules23040818

**Published:** 2018-04-03

**Authors:** Siyan Zheng, Yingpeng Zhu, Chunyan Jiao, Mengyao Shi, Lianping Wei, Yang Zhou, Qing Jin, Yongping Cai

**Affiliations:** 1 College of Life Science, Anhui Agricultural University, Hefei 230036, China; zhengsiyan_clm@163.com (S.Z.); 13856983945@163.com (Y.Z.); 15212426671@163.com (C.J.); 15103076@ahau.edu.cn (M.S.); wlpingky@163.com (L.W.); 2 Institute of Life Sciences, Jiangsu University, Zhenjiang 212013, China; zhouyang@ujs.edu.cn

**Keywords:** *Dendrobium officinale*, gigantol, response surface methodology, ultrasonic-assisted extraction

## Abstract

In order to optimize the extraction of gigantol from *Dendrobium officinale*, the influence of methanol concentration, ultrasonic temperature, and liquid ratio on extraction efficiency was analysed by the response surface analysis method. The results show that the extraction rate reached a maximum when the methanol concentration was 92.98%, the solid-liquid ratio was 27.2 mL/g, and the extraction temperature was 41.41 °C. The content of gigantol of *Dendrobium officinale* in leaves was significantly higher than that in stems, reaching 4.7942 μg/g. The content of gigantol in *Dendrobium huoshanensis* Fengdou was significantly higher than that of other species of Fengdou. This experiment has practical significance for improving the utilization rate of *Dendrobium officinale*, and provides a reference for the study of the pharmacological and biological activity of gigantol.

## 1. Introduction

*Dendrobium officinale* is a perennial herb of the orchid genus in biological taxonomy, and is commonly used as a valuable Chinese herbal medicine [1]. Medical records as early as the “Compendium of Materia Medica” show that this herb was used as a traditional medicine for relieving stomach upset, promoting body fluid production, nourishing yin, and antipyresis. There are at least one thousand species of *Dendrobium* in the world [2]. As valuable Chinese medicinal materials, *Dendrobium* species play important pharmacological roles with abundant polysaccharides, alkaloids, phenanthrenes, bibenzyls, and other biologically active substances [3]. Gigantol is able to inhibit tumour stem cell activity [4,5], and gigantol and its analogues have certain curative effects on diabetic cataracts [6]. While it was worth discussing the extraction process used in that study, the research on extraction methods and conditions of gigantol recorded in the current literature is not comprehensive.

Recent advances have allowed for the use of ultrasound technology in the food industry [7]. The effect of ultrasound is mainly attributable to a phenomenon called cavitation, which refers to the formation, growth, and implosion of tiny gas bubbles or cavities in a liquid when ultrasound is transmitted through it [8]. Extreme physical phenomena take place when the bubbles collapse at a micro-scale (1000 K and 500 MPa), and these phenomena are considered to be the cause of cell and enzyme destruction [9]. So, the extraction process of gigantol was performed in an ultrasonic cleaning bath.

Compared with response surface methodology (RSM), the traditional orthogonal design method was based on a linear mathematical model. Although it can find the best combination of multiple factors, the orthogonal design can only analyse discrete data, with accompanying disadvantages of low accuracy and poor predictability. Conversely, the response surface method, using a nonlinear model, can obtain a high-precision regression equation and a reasonable prediction about optimal process conditions. It is an extensively used method for analysis and predictions. It not only determines the interactions between the independent variables, but also reduces the number of experimental trials, development times, and overall costs. In this study, the extraction conditions of gigantol were optimized via the analysis of response surface. The three-factor and three-level central composite design based on the response surface method was used in this study to optimize the extraction method of gigantol. Moreover, the accumulation and distribution were analysed in different growth periods and tissues of *Dendrobium officinale*. The content of gigantol was also determined in the processed products of different species of Dendrobium. This study provides references for the effective development and utilization of medicinal resources.

## 2. Results and Discussion

### 2.1. Single-Factor Experiment of Gigantol Extraction

**Figure 1:**
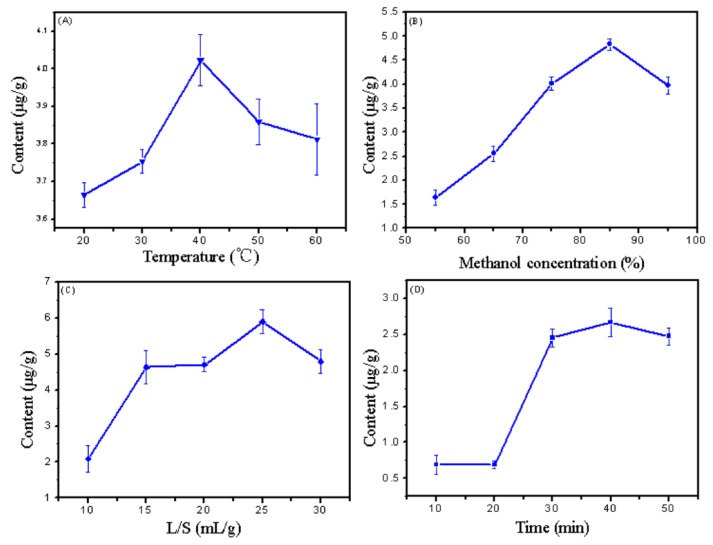
The effect of reaction conditions on the extraction efficiency of gigantol. (**A**) temperature; (**B**) methanol concentration; (**C**) liquid-to-solid ratio; (**D**) extraction time.

The effects of various factors, such as the methanol concentration, extraction temperature, liquid-to-solid ratio, and time on the extraction efficiency of gigantol were investigated by single-factor optimization experiments. The results are shown in Figure 1. According to the figure, the optimum methanol concentration was about 85%, the optimum extraction temperature was about 40 °C, the optimum liquid-to-solid ratio was about 25 mL/g, and since the extraction efficiency was not significantly changed from 30 min to 50 min, the optimum extraction time was 30 min to prevent the extract from decomposing during a long extraction [10].

### 2.2. Screening of Gigantol Extraction Factors by the Two-Level Factorial Design

Based on the results of the single-factor optimization experiment and the influence of extraction conditions, four factors affecting the extraction efficiency of gigantol were selected. The program outlined in Table 1 was used to complete data processing and analysis of variance (Table 2).

The regression model obtained by fitting the coefficient of determination *R*^2^ = 0.9768 indicates that the equation was in good agreement with the actual situation. The *p*-value < 0.05 of the model indicates that the equation was statistically significant [11]. In addition, except for time (X_4_), other factors were highly significant (*p*-value < 0.01). Thus, the methanol concentration, liquid-to-solid ratio, and temperature were chosen for further experiments of response surface.

### 2.3. Optimization of Gigantol Extraction by Response Surface

#### 2.3.1. Analysis of the Response Surface Model

The optimization of extraction conditions of gigantol was performed by using different variable combinations according to the rotatable central composite design (CCD) model in Table 3. With the aim of evaluating the suitability of the optimized model, a regression analysis and analysis of variance (ANOVA) test were completed.

Using Design Expert software to perform the quadratic polynomial stepwise regression fitting on the experimental results in Table 3, the resulting mathematical model was formed:

Y = 4.79 + 0.34X_1_ + 0.46X_2_ − 0.27X_3_ − 0.074X_1_X_2_ + 0.025X_1_X_3_ + 0.28X_2_X_3_ − 0.18X_1_^2^ − 0.28X_2_^2^ − 0.24X_3_^2^.

The experimental data were subjected to RSM, and the suitability of the model was analysed by linear regression and ANOVA (Table 4).

Model summary statistics output of gigantol showed that for different levels of methanol concentration, extraction temperature, and liquid ratio, the coefficient of determination (*R*^2^) from multiple correlation coefficients represented the relationship between the predicted and actual values in each quadratic equation. The high *R*^2^ values from the gigantol extraction efficiency model (0.8724) suggested that there was a high degree of correlation between observed and predicted values [12]. In addition, the *p*-value of the model (<0.05) indicated that the effect of this model on the results was significant. The *p*-value from the ANOVA showed the relative contribution of the model variance to the residual variance [13]. The *p*-values for X_1_, X_2_, X_3_, X_2_X_3_, X_2_^2^, and X_3_^2^ (*p* < 0.05) are shown in Table 4. The validation of the goodness of fit test was measured by using inadequate fitting. The lack-of-fit tests compared residual error with pure error from replicated design points. The *p*-value of the lack of fit from the gigantol extraction efficiency model was 0.9474. The results of lack-of-fit tests were not significant for predicted models. These results imply that this model was not significant compared to pure error, suggesting that the occurrence of such a model due to noise was unlikely [14]. Accordingly, a quadratic model was chosen to predict responses for the extraction of gigantol from Dendrobium.

#### 2.3.2. Interactions of Different Experimental Factors on the Effects of Response Variables

**Figure 2:**
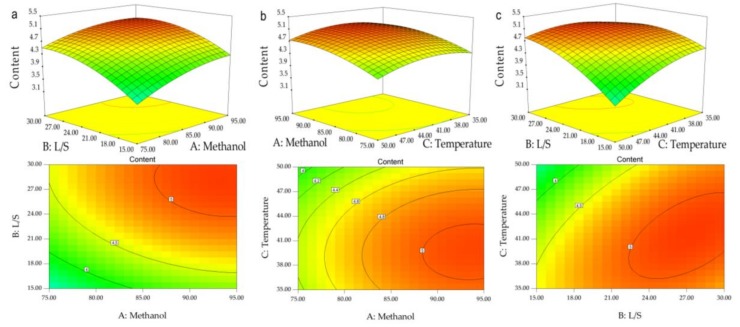
Response surface plots of the effect of factor interactions on gigantol extraction yield. (**a**) Effect of the interaction between methanol concentration and the liquid ratio; (**b**) Effect of the interaction between methanol concentration and temperature; (**c**) Effect of the interaction between temperature and the liquid ratio.

A response surface diagram is a three-dimensional spatial surface diagram of the response factors for each experimental factor, and can reflect the interaction of each experimental factor intuitively. The results showed that contour shape reflected the strength of the interaction: the more elliptical the contour shape, the stronger the interaction, and the more round the contour shape, the weaker the interaction. In order to better visualize the interactions of different experimental factor variables on response variables, three response plots and contour plots were developed (Figure 2). The influence of each factor on the response value can be visually observed from the highest point and the contour of the response surface. The more severe the response surface, the more intense the contour line, and the impact of the factors on the response value was significant. The extremum exists in the selected range, which is the highest point of the response surface, and also the centre point of the minimum ellipse of the contour.

Different interactions were visualized through a three-dimensional response surface (Figure 2). In fact, the extraction rate was calculated on the Z-axis, the other two axes represent two extraction parameters, and the rest of the extraction parameters were fixed at their intermediate levels.

It can be seen that a larger response value was obtained when the ratio of material to liquid (X2) was larger, indicating that the ratio of material to liquid (X2) had the greatest effect on the response value (Figure 2a,c). With the increase in solvent, the effective contact area of solvent and material was increased, and the solute concentration difference became larger, which was beneficial to the dissolution of gigantol, and to promote the extraction of gigantol [15]. Finally, as the amount of solvent continued to increase, the rate of extraction decreased, possibly because of the decrease in ultrasonic penetration and the lack of sufficient fragmentation of cells in the material as the solvent increased [16].

Comparison of Figure 2a,b shows the impact of the methanol concentration on extraction efficiency; at 90%, the extraction efficiency was maximum, and while the methanol concentration continued to increase, the extraction rate decreased. This may be due to a variety of different polar compounds in *Dendrobium officinale* that have different optimum solvent polarity [17], and with the increase in methanol concentration, more fat- and alcohol-soluble compositions reduced the permeability of the tissue, such that the extraction efficiency of gigantol was decreased [18].

With the change in temperature, the extraction efficiency of gigantol also showed a trend of first increasing and then decreasing (Figure 2b,c). The initial temperature increase may help the solute and solvent, in full contact, to accelerate the effective precipitation of gigantol, which may be why the heating might be softening tissues and weakening the integrity of cellular walls to improve the extraction of active compounds of plant materials [19]. The subsequent temperature increase causes a decline in gigantol extraction efficiency, probably due to the structural instability and decomposition oxidation of gigantol [20]. The extraction efficiency can also be explained by ultrasonic cavitation. With the increase of temperature, the threshold value of ultrasonic cavitation can be reduced, and the extraction efficiency can be improved. However, the continuous increase of temperature will lead to the increase of intracellular vapour pressure and introduce a cushioning effect, thus reducing the efficiency of collapse [9,21]. However, the response surface affected by the temperature was relatively gentle, indicating that the temperature changes on the extraction efficiency of gigantol were not obvious, and this result was consistent with the results of variance analysis.

#### 2.3.3. Validation of Optimal Conditions

The optimal condition was determined by a quadratic model with the variables given equal weighting. The optimal extraction condition from the model was methanol concentration 92.98%, liquid-to-solid ratio 27.2 mL/g, and extraction temperature 41.41 °C. The yields of gigantol were predicted as 5.0838 μg/g. For the practical experiment, the extraction condition employed (in five replicates) was methanol concentration 93%, liquid-to-solid ratio 27 mL/g, and extraction temperature 42 °C. A model was considered acceptable if the percentage error value was less than 10% [22]. The average experimental value for the gigantol extraction efficiency model was 5.1262 ± 0.3161 μg/g (Table 5). There was a −0.8354% difference between the predicted and observed experimental values, confirming the validity of the optimized model.

### 2.4. Analysis of Gigantol in Dendrobium

#### 2.4.1. Analysis of Gigantol in *Dendrobium officinale* Cultivation

**Figure 3:**
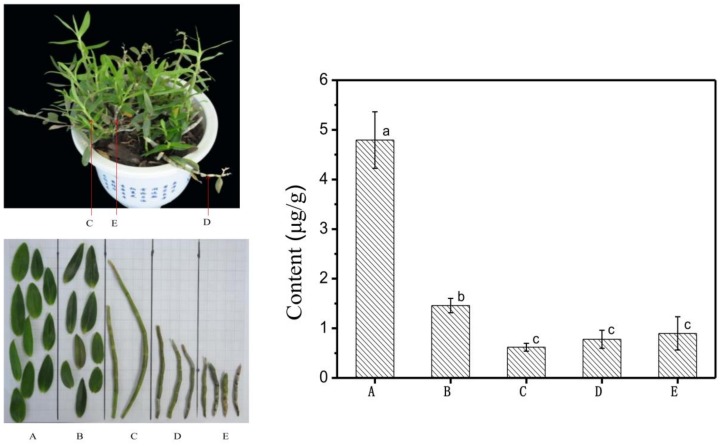
The content of gigantol in *Dendrobium officinale* at different years and in different tissues. Note: a, b and c are multiple comparison letter markings; the same letter indicates that the difference is not significant; the level of *p* < 0.01 indicates extremely significant; (**A**) annual leaf, (**B**) biennial leaf, (**C**) annual stem, (**D**) biennial stem, (**E**) three-year-old stem; the left figure is *Dendrobium officinale* cultivation and the right figure is content of gigantol.

The results showed that the gigantol content of leaves in different years was 4.7942 μg/g and 1.4603 μg/g, respectively (Figure 3), while the gigantol content of stems in different years was 0.6190 μg/g, 0.7781 μg/g, and 0.8978 μg/g, respectively. The content of gigantol in leaves decreased greatly with increasing age, while the content of gigantol in stems increased year by year. It was speculated that gigantol was first synthesized in the leaves and then transported to the stems. According to statistical analysis, the dry weight of gigantol in leaves was significantly higher than that of stems (*p* < 0.01). The dry weight of gigantol in annual leaves was 4.7942 μg/g, which was significantly higher than that of stems. Therefore, it is necessary to further analyse the accumulation and distribution of the main medicinal components in different tissues and periods of *Dendrobium officinale* to provide a scientific theoretical basis for determining the best harvest time.

#### 2.4.2. Analysis of Gigantol in Different Species of Fengdou

**Figure 4:**
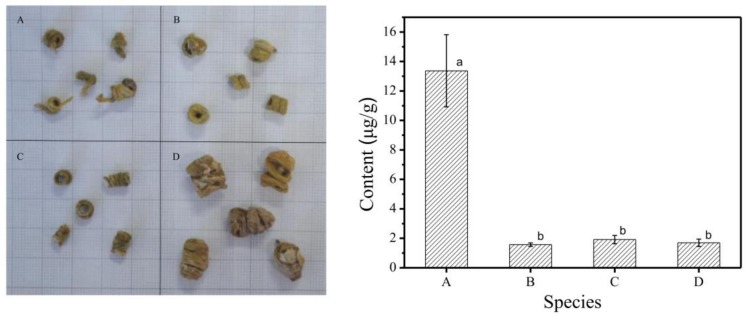
The content of gigantol in different species of Fengdou. Note: a and b are multiple comparison letter markings; the same letter indicates that the difference is not significant; the level of *p* < 0.01 indicates extremely significant; (**A**): *Dendrobium huoshanense* Fengdou, (**B**): *Dendrobium officinale* Fengdou, (**C**): *Dendrobium moniliforme* Fengdou, (**D**): *Dendrobium devoninum* Paxt Fengdou; the left figure is different species of Dendrobium Fengdou and the right figure is content of gigantol in different species of Dendrobium Fengdou.

The contents of accumulated gigantol in four species of Fengdou were determined by the above method for optimizing the extraction of gigantol (Figure 4). The results showed that the contents of gigantol in different species were 13.3639 μg/g, 1.5706 μg/g, 1.9143 μg/g, and 1.7031 μg/g, respectively. Among them, the content of gigantol in *Dendrobium huoshanensis* Fengdou—which is endemic to the Huoshan area, Anhui Province [23]—was significantly higher than that of other species of Fengdou (*p* < 0.01). The values of *Dendrobium* in Mount Huoshan were superior to other species of *Dendrobium*, according to the medicinal ingredient gigantol. The existing research suggests that both environmental and genetic factors play an important role in the synthesis and accumulation of secondary metabolites, and determine the intrinsic quality of Chinese herbal medicines. Compared with that in the stem of perennial *Dendrobium*, the content of gigantol in Fengdou was significantly higher. The reason may be that the age of *Dendrobium* Fengdou was older than that of stems, or the manufacturing process of *Dendrobium* Fengdou caused the increase of the gigantol content.

## 3. Materials and Methods

### 3.1. Herbal Samples

Tissue-cultured *Dendrobium* used in the response surface optimization research were trained by this research group of Anhui Agricultural University. Seeds of *Dendrobium officinale* were sowed and cultured in Murashige and Skoog (MS) medium. The content analysis experiment was conducted using basin-cultured *Dendrobium* seedlings grown in a greenhouse (Hefei Anhui Mulong Mountain Dendrobium Biotechnology Development Co., Ltd., Hefei, China) under the conditions of day 24 °C and night 18 °C, with natural light. The amounts of gigantol were measured in fresh stems from one- to three-year-old *Dendrobium officinale* and in fresh leaves of the annual and biennial leaves (specifically the third and fourth leaves). The plants were washed to remove impurities, and the samples were placed in a 60 °C oven drying to constant weight. Dried samples were ground to a fine powder and stored at room temperature until use [24]. Four “Fengdou” samples were purchased from herbal pharmacies in the Huoshan area of Anhui Province.

### 3.2. Chemicals and Solvents

Standard products of gigantol were purchased from the China Pharmaceutical and Biological Products Institute (lot number 111875-201202). All used chemicals, solvents, and reagents were of HPLC grade. Methanol and acetonitrile were purchased from Anhui Tedia High Purity Solvents Co., Ltd. (Anhui, China). Formic acid was obtained from Shanghai Aladdin Biochemical Technology Co., Ltd. (Shanghai, China). Pure water was purchased from the Hangzhou Wahaha Group (Zhejiang, China).

### 3.3. Extraction Procedure

Powdered samples were placed in micro-centrifuge tubes and macerated with methanol. The extraction process was performed in an ultrasonic cleaning bath (KQ5200DE, Kun Shan Ultrasonic Instruments Co., Ltd., Kunshan, China) with an output power of 80 W. The mixture was extracted in the sonication bath at a certain temperature for a specified time. The mixture was allowed to cool to room temperature. Then, the liquid phase was separated from the insoluble residue by centrifugation at 4000 rpm for 15 min. The nitrogen in the supernatant was dried and re-dissolved in 300 μL of methanol overnight. All experiments were done in triplicate.

### 3.4. Reversed-Phase-HPLC Analysis

Chromatographic analysis was carried out employing an Ultimate 3000 HPLC system equipped with an Ultraviolet–visible spectroscopy (UV-Vis) diode-array detector (DAD) (Thermo Fisher Scientific, Waltham, MA, USA). Separation was performed by using a Thermo hypersil gold C18 column (250 × 4.6 mm i.d., 5 μm particle size, Thermo Fisher Scientific). The mobile phases were prepared from 0.1% (*v*/*v*) formic acid in water (eluent A) and acetonitrile (eluent B). The gradient was as follows: 0–40 min, linear gradient from 5% to 80% B. Then, the gradient returned to 5% of eluent B and this composition was held for 10 min for re-equilibrate the column. The column temperature was set at 25 °C, the injection volume was set at 10 μL, and the flow rate was 1 mL/min. Gigantol was monitored by DAD and quantified at 279 nm. Calibration curves were calculated for gigantol on the basis of six different concentrations from 0.625 to 20 μg/mL. The calibration curve was *Y* = 3.7649*X* + 0.0481, *R*^2^ = 1, where y is the concentration (μg/mL) and x is the peak area. This formula is suitable for linearity in the range of 0.625 μg/mL to 20 μg/mL.

### 3.5. Experimental Design

Contrary to the one-factor-at-a-time optimization approach that was laborious and time-consuming, RSM was used as an ideal strategy for investigating the relationships among variables by reducing the number of experimental measurements. The extraction rate of gigantol was determined with three steps. Firstly, the single-factor experiment was carried out to estimate the optimal range of each influencing factor [25]. The effects of the methanol concentration (55–95%), extraction temperature (20–60 °C), extraction time (10–50 min), and liquid-to-solid ratio (10–30 mL/g) on the extraction rate of gigantol were investigated. The amount of gigantol was evaluated and analysed to determine the best parameters.

Secondly, several factors that had a great impact on the response values were selected by using the two-level factorial design [26,27]. Factor screening tests can estimate the main effects of factors with the least number of trials and select several factors that have significant effects on corresponding variables for further study. In this experiment, based on the above single-factor experimental results, four factors and two levels were used to filter the methanol concentration, extraction temperature, extraction time, and liquid-to-solid ratio.

Finally, the effects of the variables on gigantol extraction were investigated by using response surface methodology (RSM) [28]. The central composite design in the RSM was used in the experiment, and the second-order response surface model was obtained by fitting the experimental data. Most experimental conditions were determined and verified. Design Expert software (8.0.6 Stat-Ease, Minneapolis, MN, USA) was used to generate the experimental design, statistical analysis, and regression model for all gigantol compounds. Based on the above results, a central composite design was used to optimize independent variables of the process. All experiments in the model were done in triplicate.

## 4. Conclusions

This paper described the extraction and analysis of gigantol by RSM from *Dendrobium officinale*. Based on a single-factor experiment, RSM was applied to optimize the extraction of gigantol. The results demonstrated that the influence of the methanol concentration, liquid-to-solid ratio, and extraction temperature on the extraction rate of gigantol were not a simple linear relationship. The results of regression analysis and verification showed that this method was reasonable and feasible. The optimum extraction condition was determined to be a methanol concentration of 92.98%, a solid-liquid ratio of 27.2 mL/g, and an extraction temperature of 41.41 °C. The yields of gigantol were predicted as 5.0838 μg/g. These results deepened the extraction of gigantol from *Dendrobium officinale*.

The distribution of gigantol in different tissues of *Dendrobium officinale* has not previously been reported. By comparison, it was found that the content of gigantol in leaves of *Dendrobium officinale* was significantly higher than that in stems, and so the leaves of *Dendrobium officinale* could be used as a focus for further research of gigantol.

## Figures and Tables

**Table 1:** Two-level factorial design and results.

Run	Factor 1	Factor 2	Factor 3	Factor 4	Response
	Methanol (%; X_1_)	Liquid Ratio (mL/g; X_2_)	Temperature (°C; X_3_)	Time (min; X_4_)	Gigantol (μg/g; Y)
1	75	15	35	30	4.5111
2	75	30	35	30	4.3416
3	75	15	35	50	4.1946
4	95	30	35	50	4.0619
5	75	15	50	50	6.1446
6	95	30	50	30	6.5708
7	75	30	50	30	6.6438
8	95	15	35	50	2.5290
9	95	15	50	30	4.4223
10	75	30	35	50	6.5937
11	75	15	50	30	5.0497
12	95	30	50	50	6.2677
13	95	30	35	30	3.5000
14	75	30	50	50	8.3087
15	95	15	35	30	3.6740
16	95	15	50	50	5.1009

**Table 2:** ANOVA of the two-level factorial design.

Source	Sum of Squares	df	Mean Square	*F*-Value	*p*-Value	Significance
Model	33.22	9	3.69	28.12	0.0003	Significant
X_1_	5.83	1	5.83	44.45	0.0006	
X_2_	7.10	1	7.10	54.13	0.0003	
X_3_	14.26	1	14.26	108.62	< 0.0001	
X_4_	1.26	1	1.26	9.59	0.0212	

**Table 3:** Central composite design and results.

Run	Factor 1	Factor 2	Factor 3	Response
	Methanol (%; X_1_)	Liquid Ratio (mL/g; X_2_)	Temperature (°C; X_3_)	Gigantol (μg/g; Y)
1	95.00 (1)	30.00 (1)	35.00 (−1)	4.8982
2	75.00 (−1)	15.00 (−1)	35.00 (−1)	3.8299
3	85.00 (0)	22.50 (0)	55.11 (1.682)	3.7676
4	101.82 (1.682)	22.50 (0)	42.50 (0)	4.6509
5	85.00 (0)	22.50 (0)	42.50 (0)	5.2345
6	85.00 (0)	35.11 (1.682)	42.50 (0)	4.8230
7	85.00 (0)	22.50 (0)	29.89 (−1.682)	4.4272
8	68.18 (−1.682)	22.50 (0)	42.50 (0)	3.8907
9	75.00 (−1)	30.00 (1)	50.00 (1)	4.1397
10	95.00 (1)	15.00 (−1)	50.00 (1)	3.6150
11	85.00 (0)	22.50 (0)	42.50 (0)	4.5767
12	95.00 (1)	15.00 (−1)	35.00 (−1)	4.6886
13	75.00 (−1)	30.00 (1)	35.00 (−1)	4.1879
14	85.00 (0)	9.89 (−1.682)	42.50 (0)	3.1357
15	75.00 (−1)	15.00 (−1)	50.00 (1)	2.5093
16	85.00 (0)	22.50 (0)	42.50 (0)	4.4102
17	95.00 (1)	30.00 (1)	50.00 (1)	4.8041
18	85.00 (0)	22.50 (0)	42.50 (0)	5.2327
19	85.00 (0)	22.50 (0)	42.50 (0)	4.2105
20	85.00 (0)	22.50 (0)	42.50 (0)	5.0548

**Table 4:** ANOVA of the central composite design.

Source	Sum of Squares	df	Mean Square	*F*-Value	*p*-Value	Significance
Model	8.140	9	0.900	7.60	0.0020	Significant
X_1_	1.560	1	1.560	13.12	0.0047	
X_2_	2.840	1	2.840	23.84	0.0006	
X_3_	0.970	1	0.970	8.18	0.0170	
X_1_X_2_	0.043	1	0.043	0.37	0.5591	
X_1_X_3_	0.005	1	0.005	0.04	0.8409	
X_2_X_3_	0.630	1	0.630	5.33	0.0437	
X_1_^2^	0.470	1	0.470	3.94	0.0752	
X_2_^2^	1.160	1	1.160	9.73	0.0109	
X_3_^2^	0.840	1	0.840	7.08	0.0239	
Residual	1.190	10	0.120			
Lack of Fit	0.200	5	0.040	0.20	0.9474	Not significant
Pure Error	0.990	5	0.200			
Cor Total	9.330	19				

**Table 5:** Verification of the experimental results.

Category	Run	Yield (μg/g)		STDEV (%)
		Predictive	Experimental	
Gigantol	1	5.0838	4.6107	−0.8354
	2	5.0838	5.0319	
	3	5.0838	5.3264	
	4	5.0838	5.3549	
	5	5.0838	5.3072	
